# Fairness-aware machine learning engineering: how far are we?

**DOI:** 10.1007/s10664-023-10402-y

**Published:** 2023-11-24

**Authors:** Carmine Ferrara, Giulia Sellitto, Filomena Ferrucci, Fabio Palomba, Andrea De Lucia

**Affiliations:** Software Engineering (SeSa) Lab, University of Salerno, Salerno, Italy

**Keywords:** Software fairness, Machine learning, Empirical software engineering, Survey study, Practitioners’ perspective

## Abstract

Machine learning is part of the daily life of people and companies worldwide. Unfortunately, bias in machine learning algorithms risks unfairly influencing the decision-making process and reiterating possible discrimination. While the interest of the software engineering community in software fairness is rapidly increasing, there is still a lack of understanding of various aspects connected to fair machine learning engineering, i.e., the software engineering process involved in developing fairness-critical machine learning systems. Questions connected to the practitioners’ awareness and maturity about fairness, the skills required to deal with the matter, and the best development phase(s) where fairness should be faced more are just some examples of the knowledge gaps currently open. In this paper, we provide insights into how fairness is perceived and managed in practice, to shed light on the instruments and approaches that practitioners might employ to properly handle fairness. We conducted a survey with 117 professionals who shared their knowledge and experience highlighting the relevance of fairness in practice, and the skills and tools required to handle it. The key results of our study show that fairness is still considered a second-class quality aspect in the development of artificial intelligence systems. The building of specific methods and development environments, other than automated validation tools, might help developers to treat fairness throughout the software lifecycle and revert this trend.

## Introduction

From financial transactions to healthcare treatments, our society heavily relies on machine learning-enabled software systems (Rech and Althoff 2004). The major advances in the field of artificial intelligence allow such systems to be highly accurate and efficient, supporting—or, sometimes, replacing—humans during decision-making activities (Zhou and Chen 2018), e.g., recent applications have been successfully experimented in the context of loan management (Olson 2011) and hiring decisions (Miller 2015). However, previous work warns us to be cautious with the blind use of machine learning (Mehrabi et al. 2021). The tight reliance on historical data might indeed let a machine learning algorithm gather biased knowledge about the relations ruling a phenomenon, which might lead to unfair predictions and recommendations that, in turn, might reiterate discrimination and injustice (Barocas et al. 2017).

*Software fairness* is the branch of artificial intelligence that investigates methods and tools to reduce risks due to the misled training of machine learning algorithms (Barocas et al. 2017; Chouldechova and Roth 2020). In recent years, concerns related to machine learning fairness have also caught the attention of the software engineering research community, which is interested in providing novel engineering practices to allow practitioners to manage software fairness throughout the evolution of machine learning-enabled software systems. This interest is evident, especially in the context of the so-called MLOps, i.e., the set of practices that enable the continuous, reliable, and efficient deployment and maintenance of machine learning models in production (Biswas and Rajan 2020; Chakraborty et al. 2019; Friedler et al. 2019; Li et al. 2022; Mitchell et al. 2021; Zhou et al. 2020). Previous work demonstrated that the way machine learning algorithms are engineered has indeed an influence on the resulting level of fairness. Friedler et al. (2019) pointed out that different algorithms tend to accommodate specific formulations of fairness, while Biswas and Rajan (2020) benchmarked multiple machine learning model optimization techniques, discovering that some of them may intrinsically induce unfairness. More recent studies have faced the problem by devising novel fairness-aware approaches to train models (Li et al. 2022) and optimize learning hyper-parameters (Chakraborty et al. 2019).

In this paper, we aim at taking a further step ahead into understanding how fairness is currently engineered by practitioners and what are the gaps that the research community is called to fill to assist developers within an MLOps scenario better. In particular, we argue that a socio-technical software engineering view on the matter might be beneficial for researchers working in the field, as it might provide insights into (1) the processes applied by practitioners to deal with fairness, (2) their perception about fairness, and (3) the skills required to treat the problem appropriately. Such grounded knowledge is the paramount starting point to stimulate further research aiming at tailoring software engineering tools to the practitioners’ needs, other than devising novel project management methods to assemble teams and improve the processes applied to manage fairness.

To address the above-mentioned goals, we designed and conducted a survey study targeting professional software engineers and data scientists involved in developing machine learning-enabled software systems. We recruited 117 practitioners, inquiring them about their perception of software fairness and the practices they currently adopt to deal with it. The main findings of the study report how fairness is still considered less significant than other quality aspects, like accuracy and security. In addition, practitioners highlight that specific skills pertaining not only to the scientific treatment of the matter but also to the sociological one are required to engineer fairness-aware solutions appropriately. Last but not least, the involved professionals raised the need for analytic tools, other than novel verification and validation methods, that would be helpful to address bias gaps in machine learning engineering.

The key contributions of this paper can be summarised as follows:

1. An analysis of the practitioners’ perspective of the current processes applied to manage software fairness;
2. An overview of the limitations and challenges that the software engineering research community is called to handle to support practitioners in engineering fair machine learning systems, along with insights into the possible strategies and methods that might address those limitations;
3. A publicly available replication package (Ferrara et al. 2022), which might be used by other researchers to build on top of our findings.

### Structure of the paper

Section 2 reports the definitions of software fairness and discusses the related work in the field. In Section 3, we elaborate on the research questions driving our study and the methods employed to address them. In Section 4, we discussed the main data collection and data quality prescreening strategies, while the results of the study are discussed in Section 5. The key implications of our work are reported in Section 6, while the threats to the study’s validity are discussed in Section 7. Finally, Section 8 concludes the paper.

## Background and related work

This section introduces the problem of software fairness and the multiple definitions available so far. Then, it provides an overview of the state of the art on the matter, highlighting the main contributions to the field and the limitations that we aim to overcome with our work.

### Definitions of software fairness and related problems

Software is one of the major assets our society relies on. From business transactions to everyday tasks, people make their decisions taking into account suggestions generated by automated systems. Artificial Intelligence (AI) can be seen as a powerful tool that makes lives easier, but leveraging its recommendations also carries some drawbacks. There are many unfortunate examples in which AI solutions failed to propose useful hints, even ending up suggesting unfair decisions that damaged people by compromising their rights.

Let us consider the famed case of the Amazon recruiting software, which tended to favour men over women when recommending people to hire, prejudicing women to make a career in the company (Dastin 2018). Similar gender-related biases have been found in Google Translate, which used to hint at professions like nurse as being practiced by women, and occupations like an engineer as being held by men (Brun and Meliou 2018; Caliskan et al. 2017). Even more alarming cases of biased AI have been reported in healthcare (Obermeyer et al. 2019) and justice (Angwin and Larson 2016), where black people were deemed less healthy and more likely to commit a crime than white people.

These examples highlight the need for high-quality AI solutions able to suggest fair decisions that would not damage people’s lives and support the rise of software fairness as a new paramount quality aspect to be met by automated systems (Giovanola and Tiribelli 2023; Brun and Meliou 2018) . However, a formal standard specification of such an aspect is difficult to lay down since more than 20 definitions of software fairness have been traced out in the literature (Verma and Rubin 2018). These definitions frame the concept from different points of view and can be clustered into three main categories relying on different technical aspects, i.e., probability of predictions (a.k.a. group fairness definitions), similarity of instances (a.k.a. individual fairness definitions), and causal reasoning. Table 1 reports a summary of the different definitions of the concept of software fairness.

**Table 1:** Summary of the main categories of fairness definitions

Name	Description
Fairness definitions based on probability of predictions a.k.a. group fairness definitions	
Group fairness, a.k.a. statistical parity	According to this definition, a binary machine learner is fair if individuals of a protected group defined based on a protected attribute are associated to the positive class with the same probability of assigning the individuals of non-protected groups.
Predictive Parity a.k.a. outcome test	According to this definition, a binary machine learner is fair if both protected and non-protected groups have the same probability to assume the real value of classification, i.e., the probability of individuals to be classified as true positive/negative is the same.
False positive error rate balance a.k.a. predictive equality	According to this definition, a binary machine learner is fair if the probability of individuals being associated with the *positive* class even though the real value of classification is opposite is the same for both protected and non-protected groups, i.e., the probability of being a false positive is the same.
False negative error rate balance a.k.a. equal opportunity	According to this definition, a binary machine learner is fair if the probability of individuals being associated with the negative class even though the real value of classification is opposite is the same for both protected and non-protected groups, i.e., the probability of being a false negative is the same.
Equalized odds a.k.a. disparate mistreatment	This definition combines the previous two (predictive equality ad equal opportunity), considering a binary classifier as fair if both, the protected and unprotected groups have the same rates of true positive and false positive instances.
Fairness definitions based on similarity of instances a.k.a. individual fairness definitions	
Causal Discrimination	According to this definition, a binary machine learner is fair if it produces the same classification for any two subjects with the same attributes. For instance, this implies that male and female applicants who otherwise have the same attributes will either both be hired or not.
Fairness through unawareness	According to this definition, a binary machine learner is fair if no sensitive attributes are explicitly used in the decision-making process.
Fairness through awareness	According to this definition, a binary machine learner is fair if similar individuals obtain similar classification results (It is the more elaborated and generic similarity definition of fairness).
Fairness definitions based on the concept of causal reasoning	
No unresolved discrimination	According to this definition, a binary machine learner is fair, if the relative causal dependency graph does not present any path among sensitive attributes and learner results.
Counterfactual fairness	According to this definition, a binary machine learner is fair, if the relative causal dependency graph does not present any *indirect* path among sensitive attributes and learner results.

Reference: Verma & Rubin: Fairness Definitions Explained - 2018 (Verma and Rubin 2018)

Definitions based on similarity of instances describe an AI solution, i.e., a machine learner, as being fair if (1) sensitive attributes—like gender—are not used in the decision-making process, and (2) individuals with the same attributes are assigned to the same class. For instance, a woman and a man having the same set of attributes should both be considered suitable for hiring or not. This set of definitions is quite similar to the ones based on the concept of causal reasoning, which drives machine learners to classify individuals with respect to cause-effect relations holding between their attributes. Under this point of view, an AI solution is considered fair if its predictions do not causally depend on any protected attributes. Given all the nuances depicting the theory of software fairness, it is not clear how this quality aspect is, and should be, treated in practice.

### State of the art

Software systems are being affected by new kinds of vulnerabilities, generated by their (in-)ability to operate impartially and without ethical biases, i.e., guaranteeing fairness (Brun and Meliou 2018; Chakraborty et al. 2021). These new vulnerabilities are strictly related to the concept biases affecting the learning algorithms and training data that are necessary to develop an AI-based software solution. However, they can be caused by many other aspects, such as requirements leakages, poor design choices, development bugs, or wrong interactions between components (Brun and Meliou 2018). Horkoff claimed the need for ethically correct machine learning (ML) development, introducing fairness as a basic quality aspect for every ML module (Horkoff 2019). For these reasons, software fairness has captured the attention of both the research communities of Artificial Intelligence and Software Engineering, which set up the main goal of guaranteeing fair AI solutions.

Finkelstein et al. (2008) first introduced the concept of fairness in the requirements analysis and optimization process. They took into account the various facets of defining a system as fair from an ethical point of view, highlighting that some slight differences among the definitions of software fairness are in contrast with each other. Therefore, they framed the problem as a “search-based optimization” task, to find a compromise among different fairness definitions in various contexts. Along these lines, further approaches proposed novel algorithms to deal with fairness measurement constraints on data and learning tasks. For instance, Celis et al. (2019) introduced a meta-algorithm able to create fair classification models across different fairness-specific constraints, showing promising results in terms of fairness and accuracy.

Identifying the fairness requirements of a software system is just the first step toward guaranteeing unbiased decisions. Brun and Meliou outlined the challenges in the development of a fair ML solution and drafted suggestions to be followed during each phase of the software life cycle (Brun and Meliou 2018).

In particular, to avoid discrimination, it is crucial to adopt accurate design practices and fairness-focused algorithms and to conduct specific fairness testing activities. The most relevant causes of unfairness lie in training datasets and code and may be due to underlying biased assumptions that people make during development (Vasudevan and Kenthapadi 2020). Several studies investigated the relations between data and outcomes to understand the reasons behind the unfair behaviour of machine learning systems. According to Zhang and Harman (2021), the problems with software fairness rise from the specific application domains and the features used to describe individuals. They conducted an empirical investigation using different datasets and fairness metrics and found that a higher number of features can help increase the fairness level of an application. Hort and Sarro (2021), instead, introduced the concept of *anti-protected attributes*, i.e., attributes of a dataset, different from the sensitive ones, that might improve fairness if their influence on the model’s outcome is higher than other attributes of the training dataset. Other studies exploit the concept of explainable artificial intelligence to provide a rationale behind dependencies among sensitive features and fairness of the learning models (Menzies et al. 2021; Chakraborty et al. 2021).

Beyond understanding the relations between data and outcomes, researchers have been proposing different solutions to manage and mitigate models’ discrimination. These strategies can be categorized according to the machine learning pipeline stage where they can be applied:

#### Fair data selection and preparation

Moumoulidou et al. (2020) observed that the training data selection is not a trivial process under fairness constraints and experimented with the creation of different training subsets. They proposed a fairness-oriented variant of the Max-Min diversification algorithm and proved it is an NP-Complete task. They designed approximation algorithms to deal with the problem of properly selecting training data to avoid discrimination biases.

Several data balancing strategies have been proposed in the literature, to build a bias mitigation system starting from pre-processing activities. Calmon et al.’s optimized pre-processing for discrimination prevention (Calmon et al. 2017), Chakraborty et al.’s Fair-SMOTE (Chakraborty et al. 2021) is based on the idea that the main causes of discrimination biases are the labels assigned to the features used for the training of the machine learning model. Peng et al. proposed FairMask (Peng et al. 2023), a model-based pre-processing method that mitigates biases by applying data balancing based on the explanation of the root causes of unfairness. Biswas and Rajan (2021) observed that the majority of fairness issues pertain to the training data. As such, they assessed multiple *fair-data transformation* patterns against 37 different machine learning pipelines, elaborating on the strengths and weaknesses of each pattern.

#### Fairness-aware model design and building

Other works proposed different approaches to manage fairness as an in-processing task. Hort et al. (2021) proposed Fairea, a mutation approach to benchmark and quantify the fairness-accuracy trade-off by using formal bias mitigation methods. Recently, Chen et al. (2022) compared the performance of Fairea and Fair-SMOTE against MAAT, a novel ensemble approach that combines different learning models with the same goal in order to improve the fairness-performance trade-off of a machine learning algorithm. authorname (2022), instead, conducted an empirical evaluation of fairness in the context of neural networks. They proposed a novel causal analysis method to understand the distribution of sensitive inputs among the input and the hidden neurons of a network. Other relevant studies focused on the concepts of adversarial learning and generative adversarial networks to achieve both high accuracy and high levels of fairness of the model under training (Xu et al. 2019; Zhang et al. 2018).

#### Fairness-aware model evaluation and testing

Insights into a system’s fairness level can be gained through testing and validation. Galhotra et al. (2017) recently proposed the Themis approach to assess whether and how machine learning-based solutions tend to be biased. For each feature in the dataset, Themis generates test suites to compute the level of discrimination that the system applies concerning it, addressing the problem of finding the root causes influencing the unethical behaviour of the software. Differently, Soremekun et al. (2022) proposed ASTRAEA, a context-free grammar-based testing approach that exploits grammar-based mutations to generate test inputs and oracle, detecting fairness violations by using anomaly detection. Similarly, Perera et al. (2022) proposed *fairness degree*, a search-based testing approach for regression-based machine learning systems. Udeshi et al. (2018), instead, proposed AEQITAS, an automated fairness testing approach that adopts a systematic perturbation approach to discover discriminatory inputs and exploit them to generate a synthetic augmentation of the original training set to retrain the model to mitigate its unfair behaviour. Other specific studies related to fairness testing relied on specific machine learning strategies, e.g., explainable AI and combinatorial analysis (Aggarwal et al. 2019; Patel et al. 2022).

#### Through a broader lens

Several studies designed fairness-aware pipelines to drive the development of model fairness among different stages of the learning process. For instance, Chakraborty et al. (2020) proposed Fairway, an integrated pipeline that combines preprocessing and in-processing strategies to remove ethical bias from both training data and built models. Other studies (Johnson and Brun 2022; Bellamy et al. 2019; Bantilan 2018) contributed with novel libraries that may assess fairness throughout the learning process.

Some literature reviews and survey studies had research objectives close to ours. For the sake of clarity, Table 2 overviews the main similarities and differences with respect to our research.

**Table 2:** Summary of the main secondary studies on Software Fairness

Secondary study	
Mehrabi et al., A survey on bias and fairness in machine learning (Mehrabi et al. 2021)	
Summary:	Similarities and Differences:
They analyzed the existing literature and defined two taxonomies of (1) the most common fairness and bias definitions, and (2) the state-of-art strategies that researchers proposed to mitigate unfair outcomes in different machine learning application domains.	–Similar objective: Investigate and generalize knowledge on the treatment of fairness in terms of definitions, metrics, strategies and causes of bias;
	–Different method and target context: We perform a large-scale survey study involving practitioners working on ML-Intensive systems.
Pessach and Shmueli, A Review on Fairness in Machine Learning (Pessach and Shmueli 2022)	
They performed a systematic literature review focusing on classification tasks and discussed trade-offs between fairness and model accuracy, categorizing fairness-enhancing mechanisms in pre-processing, in-preprocessing, and post-processing approaches, depending on when they should be applied.	–Similar scope: Investigate and systematize knowledge about fairness treatment strategies, metrics, and trade-offs;
	–Different method and goal: We are interested in understanding how fairness is perceived and treated by practitioners with respect to other six non-functional requirements, by performing a large-scale survey study.
Pagano et al., Bias and Unfairness in Machine Learning Models (Pagano et al. 2023)	
They conducted a systematic literature review to collect the most used datasets, metrics, techniques, and tools to detect and mitigate bias.	–We rely on the findings of Pagano et al. to understand the available tools, and we assess whether they are currently leveraged in the practice.
	–Different method and target context: We perform a survey study involving practitioners.
Le Quy et al., A survey on datasets for fairness-aware machine learning (Le Quy et al. 2022)	
They analyzed datasets provided in the literature to investigate the relationships between protected attributes and class attributes via Bayesian networks.	–Different method and granularity: We conduct a large-scale survey study and collect practitioners’ insights on the common causes of bias in working projects.
Bird et al., *Fairness-Aware Machine Learning [...]* (Bird et al. 2019)	
They drew an overview of the lessons learned in the literature and provided the community with a research road map toward a *fairness-first* approach, a new development way to manage fairness since the first stages of a typical machine learning development process.	–Different method and goal: We perform a survey study to collect practitioners’ opinions that we believe will be useful to formulate theoretical fairness-oriented development frameworks.
Xivuri and Twinomurinzi, A Systematic Review of Fairness in AI Algorithms (Xivuri and Twinomurinzi 2021)	
They analyzed 47 papers to understand how the research community dealt with fairness in terms of method, domains, practices, and locations. They highlighted how fairness is currently more focused on technical and social/human aspects rather than the economic ones, and how most studies have been conducted in Europe and North America.	Similar broadness: We consider different geographical locations, application domains, people’s levels of experience and backgrounds, roles and job responsibilities;
	Different method and target context: We perform a survey study involving practitioners.
Shrestha and Das, Exploring gender biases in ML and AI academic research [...] (Shrestha and Das 2022)	
They analyzed 120 papers in the context of a systematic literature review on gender bias in machine learning and artificial intelligence, warning that gender-related biases are less explored and require more attention by the research community.	–Different method and scope: We perform a large-scale survey study to understand in which phases of a typical machine learning pipeline the practitioners adopt specific strategies to deal with all kinds of bias and ethical issues.
Catania et al., Fairness & friends in the data science era (Catania et al. 2022)	
They analyzed the existing literature to assess how researchers investigated unethical behaviour of data-driven automated decision systems in the context of complex data science pipelines.	–Different method and goal: We conduct a survey with practitioners to gain understanding of how fairness is treated in the practice, throughout the whole development process.
Madaio et al., Assessing the Fairness of AI Systems [...] (Madaio et al. 2022)	
They conducted semi-structured interviews and structured workshops with 33 AI practitioners to understand their perspectives on processes, challenges, and needs in the machine learning system development process.	–Similar target context: We are interested in grasping the practitioners’ perspectives on the state of the practice;
	–Different method and generalizability of results: We perform a large-scale survey study involving respondents with variegated backgrounds.
Fabris et al., Algorithmic Fairness Datasets: the Story so Far (Fabris et al. 2022)	
By surveying the literature, they developed a structured ontology of more than 250 datasets, that have been employed for different fairness-critical tasks in over 30 different application domains.	–We leverage Fabris et al.’s findings to design our research materials, i.e., the survey questions, considering the fairness-critical application domains listed in the ontology, and providing the participants with the possibility to list additional context they work in;
	–Different method and goal: We conduct a survey to understand the resources and strategies involved in a fairness-critical development scenario.
Saha et al., Measuring non-expert comprehension of ML fairness metrics (Saha et al. 2020)	
They proposed a metric to represent the non-experts’ comprehension of specific statistical fairness definitions, exploring the relationship between comprehension, sentiment, demographics, and the definitions themselves. They validated the metrics via an online survey with non-expert participants, to test its reliability over three specific fairness statistical definitions, i.e., demographic parity, equal opportunity, and equalized odds.	–Similar method: We administered an online survey to involve industrial practitioners;
	–Different goal and target context: We surveyed experts practitioners with experience on fairness-critical machine learning projects to collect information on multiple aspects, i.e., the clarity, usefulness and applicability of different fairness notions, and how fairness is relevant with respect to other non-functional attributes in a typical industrial context.

### Considerations on the State of the Art

The work discussed above covered different angles of the problem of software fairness, sharing the goal of formalizing definitions and methods to avoid discrimination and biases in machine learning-based applications. While great effort has been spent in proposing solutions to the problem, little is known about the actual employment of such solutions in realistic working environments. Several secondary studies provided a broader view of the most common definitions, datasets, strategies, and approaches to deal with machine learning fairness, which the research community formalized in the last few years. However, only a few studies took into account the practitioners’ perspective.

We point out a missing overview of the state of the practice that could highlight the practitioners’ needs and drive the research community toward the definition of even more relevant solutions applicable in real development scenarios. Moreover, we notice that most research on the matter is focused on data handling strategies, and few works consider other phases of the software life cycle. However, fairness has recently been deemed a paramount quality aspect, and should be treated carefully as any others (Giovanola and Tiribelli 2023; Brun and Meliou 2018).

#### Our contribution

We believe that the first step toward the proper treatment of fairness requirements in real working environments consists in assessing the current state of the practice to identify strengths and weaknesses and propose novel solutions. Our work aims at providing insights into the point of view of practitioners involved in the development of ML-based software solutions. Differently from the previous empirical studies on the matter, the main objectives of our investigation are focused on providing useful large-scale insights about practitioners’ perspectives on the understandability and applicability of definitions, strategies, and approaches to deal with machine learning fairness, that research community proposed in the years. In addition, we are interested in facing this problem from a Software Engineering point of view, trying to link, for the first time, the main characteristics of machine learning fairness with the aspects that typically characterize a software engineering development process, i.e., understanding how fairness is perceived with respect to other machine learning non-functional aspects, what are the strategies and instruments that practitioners consider to deal with fairness in a typical ML development life cycle, and what are the working figures that should be involved in a fairness-oriented development team.

In particular, our contribution depicts an overview of (1) whether fairness is considered to be a critical quality aspect by practitioners, (2) how it is treated in each phase of the software life cycle, and (3) who are the subjects responsible for its management. We are convinced that our findings can drive future work toward better strategies for dealing with fairness, starting from the needs of the current situation.

## Empirical study design

In this section, we present the research questions driving our research and the methods employed to address them.

### Research questions

The ultimate *goal* of this study was to understand the practitioners perspective on software fairness in the *context* of the development of ML-Intensive systems, with the *purpose* of gaining insights into the state of the practice to drive future work and make fairness a real first-class quality aspect (Brun and Meliou 2018). The *perspective* was of both researchers and practitioners: the former are concerned with the observation of the current situation to formalize needs, strategies, and processes to deal with fairness; the latter are interested in obtaining proper suggestions on how to consider the ethical aspects of a software system.

Our study has been driven by several limitations emerging from the state of the art. First and foremost, we observed that the concept of software fairness itself could be defined in different ways (Verma and Rubin 2018). We noticed the lack of a clear overview of practitioners the opinions on the most suitable definitions and practices to follow in real scenarios. Thus, we asked:

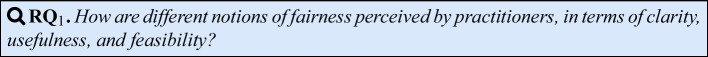



Software fairness should be treated as a crucial quality aspect in machine learning development (Brun and Meliou 2018). However, in a real development scenario, it is not affordable to perfectly meet all the requirements, and trade-offs must be taken into account to deliver the software in a timely and adequate way (Finkelstein et al. 2008). The ethical appropriateness of the system should be considered as one of the top-priority aspects to fulfill, but it is unclear whether it is deemed relevant by practitioners. Thus, we formulated our second research question:

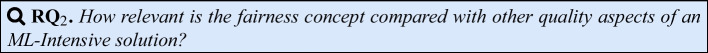



In an ML-Intensive software life cycle, a number of additional phases supplement the classic development, i.e., data engineering for model training, testing, and validation. In each phase of the development, specific precautions and solutions should be applied to guarantee that fairness requirements are met (Galhotra et al. 2017; Islam et al. 2021; Moumoulidou et al. 2020). Given the number of approaches proposed in the literature to deal with fairness, we asked:

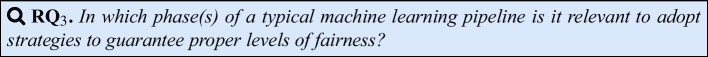



Given the recent emergence of the topic, we acknowledged that software fairness is characterized by a lack of a standard engineering process for its definition and management, unlike other quality aspects (Brun and Meliou 2018). In this context, different professional figures take part in the development of an ML-Intensive software solution, i.e., Data Scientists, Software Engineers, Data and Machine Learning Engineers, Project Managers, and Software Analysts. The individual and collaborative responsibilities that each role holds on guaranteeing fairness, as well as the existence of a dedicated *Fairness Expert*, are currently unknown. Therefore, we asked:

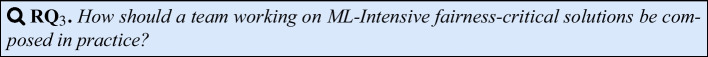



Through this set of research questions, we finally aimed at enlarging the current body of knowledge on machine learning fairness, providing insights into some of the key software engineering practices that practitioners employ when addressing fairness in real-world software systems.

### Research method and study variables

**Fig. 1:**
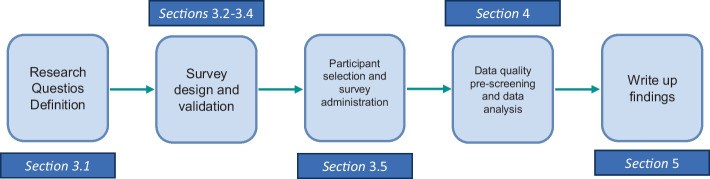
Study research method

To gather insights from practitioners, multiple empirical research methods could be exploited, e.g., large-scale survey questionnaires, structured interviews, literature reviews, or data aggregation of major trends from industrial case studies (Seaman 1999). Among the various options, we opted for a *survey study*. In particular, while qualitative studies conducted with a small sample of participants, e.g., semi-structured interviews, aim at collecting finer-grained, specific experiences of the interviewees that may later be transferred to a larger population of practitioners, survey studies have the goal to collect knowledge from a larger sample in an effort of finding common patterns related to the management of a certain phenomenon. The goal of our work is to understand the state of the practice: in this sense, a larger sample may provide more insights into the matter and enable the identification of patterns that are verified independently from the specific application domain, development team, etc., hence going toward an improved understanding of the general state of the practice of machine learning fairness. Figure 1 overviews the main methodical steps adopted to address the goals of our research, along with the reference to the sections of the article discussing them.

First, for each research question, we defined the empirical study variables, i.e., the independent and dependent variables involved, other than the confounding factors and treatments administered to participants.

- *Variables involved in RQ*_1_. Our first research question was aimed at understanding how the notions of fairness are perceived by practitioners. Therefore, the independent variable was the *notion of fairness*, a categorical variable assuming three values, i.e., the main kinds of definitions provided by previous literature (Verma and Rubin 2018). We identified three dependent variables involved in this research question, i.e., the *degree of clarity*, *usefulness*, and *feasibility* of the definition.
- *Variables involved in RQ*_2_. Since RQ_2_ pushed us to understand the relevance of fairness compared to other quality characteristics, we identified such *quality aspects* as the independent variable and the compared *relevance of fairness* as the dependent variable. We recognized that the relevance of fairness is dependent on the *application domain* it is considered into; therefore, we identified it as an independent variable to take into account, to gain a detailed understanding of how fairness is deemed important in different contexts.
- *Variables involved in RQ*_3_. Our third research question was targeted at understanding in which phases of an ML pipeline is it relevant to adopt strategies and employ tools to guarantee proper levels of fairness. The investigation driven by such a question was three-fold. Firstly, we were interested in comprehending in which phases of the pipeline is it relevant to take action; therefore, the first independent variable we identified was the pipeline stage, and the dependent variable consisted in the extent to which is relevant to take into account fairness in the stage. Secondly, we wanted to assess what are the strategies and tools currently employed in the state-of-the-practice; hence, we considered the available tools as the independent variable and the extent to which they are used in practice as the dependent variable. Lastly, we were concerned about understanding in which specific phases of the ML pipeline the tools are actually useful. In this third part of the investigation, the tools again acted as the independent variable, and the phases in which is useful to employ the tools represent the dependent variable.
- *Variables involved in RQ*_4_. As RQ_4_ drove us to understand the desirable composition of a team working on ML-Intensive fairness-critical solutions, it was worth investigating (1) which professional roles should be involved in each phase of the development, and (2) which collaborations among roles are crucial to guarantee proper levels of fairness. Therefore, in the context of the first part of the investigation, we identified the professional role and the pipeline phase as the independent variables, and the importance of leveraging the professional role in the phase as the dependent variable. For the second part of the investigation, we considered the pairs of professional roles as the independent variable, and the relevance of the collaboration between roles as the dependent variable.
- *Counfounding factors.* For all research questions, the confounding factors were represented by the practitioners’ education, company size, and level of experience with fairness.
- *Treatment.* Our study included one treatment, namely the administration of a survey to gather information on dependent variables and confounding factors.

The empirical study variables informed the subsequent data collection and analysis strategies, as discussed in the remainder of the section. We reported a detailed spreadsheet regarding the principal variables and co-factors of our analysis in the online appendix of this paper (Ferrara et al. 2022).

**Fig. 2:**
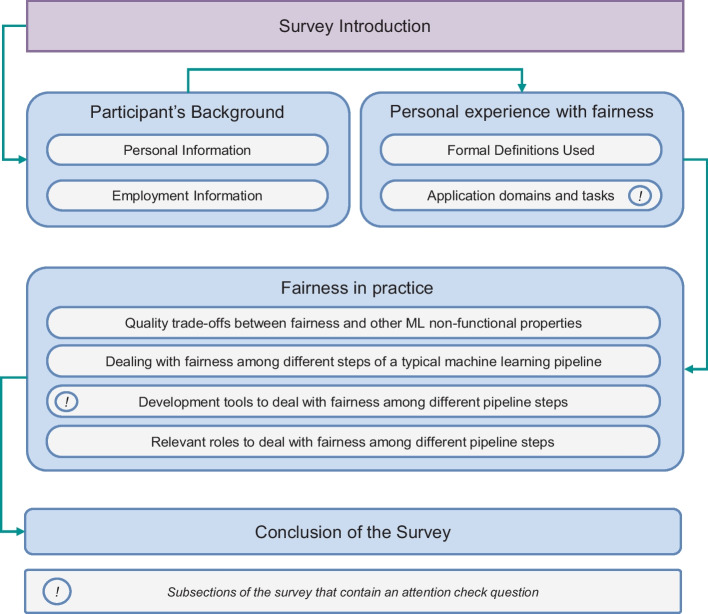
Structure of the Survey

**Table 3:** Relation between RQs and survey

Sub section	Topic	Relative RQs
Section - Survey Introduction		
Welcome Page	Explain the main topics of the study, introduce the research team, and gather consent to treat and process personal data.	-
Section - Participant’s Background		
Personal Information	Gather personal and demographic information for data analysis.	RQ1, RQ2, RQ3, RQ4
Employment Information	Collecting working experience data for data analysis.	RQ1, RQ2, RQ3, RQ4
Section - Personal experience with fairness		
Formal Definitions Used	Surveys practitioners’ opinions and expertise with different notions of fairness	RQ1
Application domains and tasks	Collect information about individuals’ expertise in fairness treatment across various application domains and machine learning tasks.	RQ1, RQ2, RQ3, RQ4
Section - Fairness in practice		
Quality trade-offs between fairness and other ML non-functional properties	Evaluate the relevance of fairness in comparison to other non-functional aspects of machine learning quality across different application domains.	RQ2
Dealing with fairness among different steps of a typical machine learning pipeline	Collect information about the importance of addressing machine learning fairness across different phases of a well-defined machine learning pipeline.	RQ3
Development tools to deal with fairness among different pipeline steps	Gather insights on well-known development tools and resources used to manage fairness in different steps of a machine learning pipeline.	RQ3
Relevant roles to deal with fairness among different pipeline steps	Gather information on the relevance of fairness and the need for collaborations among various stakeholders in different stages of a machine learning pipeline.	RQ4
Section - Conclusions		
Greetings and Future Research(s) Invitation	-	-

### Survey design

To balance the need to have a reasonably short survey and the necessity to gain enough information to answer our research questions, we designed our survey following the guidelines by Kitchenham and Pfleeger (2002); Andrews et al. (2003), and Wohlin and Runeson (2012). n particular, we made sure to (1) use a clear, unambiguous, and concise vocabulary to avoid confusion in the participants, (2) include both closed-ended and open-ended questions, with the former simplifying the analysis of the results and identification of general patterns, while the latter allowing the extraction of finer-grained insights into the participants’ perspective, (3) insert attention checks and alternative flows, to automatically discard answers obtained from distracted respondents. We grouped the survey questions into sections to logically separate the kinds of insights that we requested the participants to share. For the sake of conciseness, in this paper, we present an overview of the information that we asked for, reporting that the complete set of questions is available in the online appendix of this paper (Ferrara et al. 2022). Figure 2 depicts the survey structure, while Table 3 reports how each of the survey sections is related to our research questions.

#### Survey Introduction

To welcome the participants, we introduced them to the research problem through a brief introductory text and an example of discrimination. We did not provide any definition of fairness: one of our goals was indeed understanding the practitioners’ perception of the definition of fairness used in practice; therefore, providing them with a definition might have led to the introduction of a bias. The introductory part ended with information about us, the way we would have treated personal data, and the request to provide consent to the participation and dissemination of the collected data, in aggregate form, within a research paper.

#### Participant’s Background

. The first section of the survey was focused on obtaining information about the participant’s background, such as demographic aspects, level of study, and employment positions. In this section, we also asked the participants whether they have ever worked in machine learning development and in projects where fairness was considered a non-functional requirement to satisfy: in case of negative responses, the participant’s submission was not considered in the scope of data analysis, as they could not provide any feedback about their experience with fairness in practice.

#### Practitioner’s experience with fairness

This section aimed to investigate the practices employed in fairness-critical development by assessing the practitioners’ specific expertise in dealing with machine learning fairness. We first asked them to point out, according to their working experience, in which learning tasks and application domains they dealt with fairness—to propose suitable learning tasks and application domains related to fairness, we relied on an existing ontology that comprehensively classified those pieces of information (Fabris et al. 2022). Afterward, we inquired participants about the formal notion(s) used to define fairness in their past working experience. In addition, we asked to evaluate the categories of fairness notions (Verma and Rubin 2018) in terms of their (1) *clarity*, i.e., how clear and understandable are fairness notions in a certain category, (2) *usefulness*, i.e., how helpful are the definitions within a certain category for managing and improving fairness in practice, and (3) *feasibility*, i.e., how difficult is it to implement a particular category of fairness notions in practice.

**Table 4:** Main quality aspects compared to fairness

Quality aspect	Definition
Usability	The ability of a machine learning module to provide appropriate conditions for system users to perform the tasks for which it was designed.
Reliability	The probability with which a machine learning module executes its tasks over time for a specific number of users without failure.
Performance	The ability of a machine learning module to perform actions considering well-defined speed and time constraints.
Accuracy	The level of data points correctly predicted by a machine learning module, compared to the total number of predictions made.
Security & Safety	The ability of the learning module to detect the risks of malicious attackers potentially damaging the system and prevent failures or vulnerabilities that make the iter system potentially dangerous.
Maintainability & Retraining	The degree to which a machine learning module can be modified to be adapted to changes in the usage environment or can be re-trained with a new training set resulting from changes in the usage environment.

When presenting the various fairness notions to practitioners, we explicitly referred to those originally proposed by Verma and Rubin (2018), other than explaining each notion as done by the reference study, i.e., by presenting what that notion would imply in a real-case scenario. As an example, when presenting the *similarity notions of fairness*, we provided the following description:

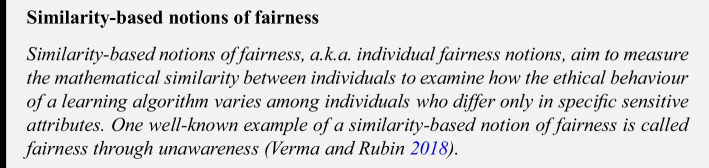



In addition, we asked practitioners to further elaborate—through a follow-up open-ended question—on any concrete application examples of those fairness notions they have experienced in the past.

In terms of presentation of fairness notions, it is worth explaining the rationale behind our choice, i.e., presentation based on formal definitions, rather than the presentation of concrete examples of those notions applied to fairness-critical systems. While such a concreteness would have had some benefits, e.g., participants might have been more aware of the *specific* use case scenario that they were supposed to assess, it may have also biased participants toward replying keeping that specific use case scenario in mind. We believe that concreteness, in this case, has more risks than benefits. Our study targets practitioners who are experienced in dealing with machine learning fairness in multiple application domains: by reporting a concrete case related to a specific application domain, we could have possibly led practitioners specialized in other contexts to address the specific questions by reporting experiences connected to their own application domain. This would have been a notable source of bias, as the insights collected might have not or only partially reflected the actual perspective of the practitioners involved. On the contrary, our work aims at eliciting patterns that may be verified under multiple application domains. For this reason, we opted for a different presentation strategy.

**Fig. 3:**
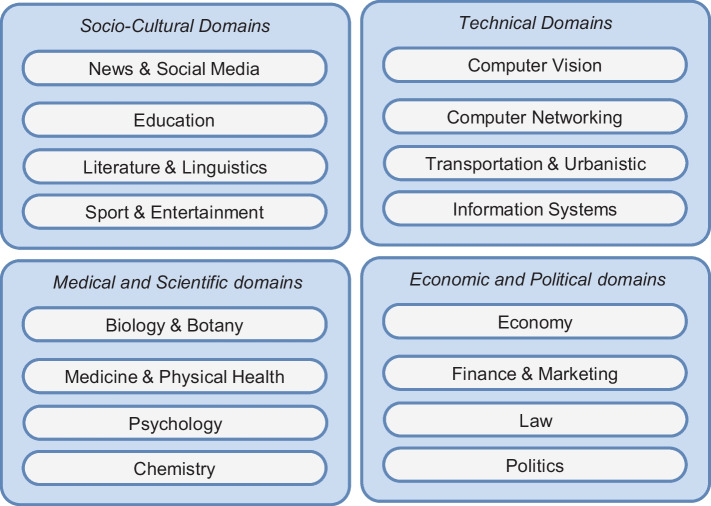
Well-known fairness-critical application domains

#### Fairness in practice

In this section, we first assessed how relevant fairness is compared to other quality aspects—Table 4 reports the quality aspects considered. In doing so, we had to deal with a potential threat to validity, i.e., practitioners might be less confident to report their perspective when assessing application domains far from their expertise. We, therefore, clustered 16 different fairness-critical application domains into four groups, asking practitioners to address the question only for those clusters with which they felt more confident. Figure 3 reports the clusters defined.

Afterward, we investigated in which development phase(s) or process(es) fairness should be treated. Questions connected to this matter were formulated according to the steps included within an MLOps pipeline (Hapke and Nelson 2020; Studer et al. 2021; Zhou et al. 2020), i.e., *“Data Engineering”*, *“Model Engineering”*, (3) *“Model Performance and Quality Monitoring”*, and *“Data Analysis and Experimentation”* (Kolltveit and Li 2023).

In addition, we asked which commercial or research tools practitioners felt useful throughout the MLOps pipeline. Finally, we also aimed at collecting information about the professional roles and collaborations thereof required for engineering fairness within an MLOps pipeline.

#### Conclusion of the survey

Before thanking the participant, we allowed them to enter their email address to (1) receive a summary of the results and/or (2) participate in possible empirical investigations, like future follow-up interviews.

#### Ethical considerations

It is important to remark that in our country, it is not mandatory to seek approval from an Ethical Review Board when releasing surveys involving human subjects. However, when designing the survey, we took into account many possible ethical and privacy concerns (Hall and Flynn 2001). To mitigate such issues, in the introductory text of the survey we informed the participants about the following precautions.

- We invited participants to share information that can be covered by strict business restrictions, so we remarked that the survey compilation could be left at any time, nullifying the final submission;
- We guaranteed the practitioners’ privacy, without using the collected information, if not for the explicit goals reported in the starting section of the survey. In any case, all direct references to people were anonymized before the analysis of the results;
- Participants were not obliged to share with us any of their sensitive business information. For this reason, we always provided participants with the chance of selecting the *“Prefer not to say”* option in every question;
- Questions asking for potentially sensitive information, e.g., gender or age, were all made optional.

### Survey validation

Before running the survey, we performed a pilot study (Andrews et al. 2003; Morin 2013), i.e., an experiment with a small sample of trusted participants who can provide feedback on the survey’s length, clarity, and structure. In our case, the pilot study was conducted by five Ph.D. students of the University of Salerno with expertise in the fields of *Software Engineering for Artificial Intelligence*, *Machine Learning Fairness*, and *Empirical Software Engineering*. The participants had expertise in designing and developing research solutions to deal with non-functional aspects of machine learning systems and in conducting quantitative and qualitative empirical investigations with industry practitioners. In addition, one of them had also previous industrial experience—he worked as a data scientist in an Italian IT company for three years. The sample considered as part of the pilot study intrinsically differs from the population targeted by the survey study. This may raise some potential threats to validity due to the misalignment between the pilot testers and the target population. Nonetheless, it is worth clarifying that the pilot study had the primary goal to verify the expected completion time and the understandability of the questions; on the contrary, it was not aimed at assessing any technical competencies required to fill in the survey. As such, it is reasonable to believe that the reliance on Ph.D. students had a contained impact. In addition, the pilot testers involved have expertise that may resemble those of the target population: as a consequence, the selection of pilot testers has been done to reduce the gap between the pilot testers and the actual target population of the study. Yet, we still acknowledge a potential threat, which we elaborate further in Section 7. .

The main issues raised in the pilot study were concerned with (1) the clarity of the terminology used when referring to technical concepts and definitions; (2) the phrasing of the questions related to the evaluation of the fairness trade-offs, which was deemed too complex to understand; (3) the clarity of the study objectives and the data management policies; and (4) typos and/or grammatical errors. This time the pilot experiment did not highlight any redundant questions - this is likely due to the fact that the survey design was informed by the original study. In any case, we took the input of the pilot testers into account to modify the survey study before running it on a large scale. Specifically, we modified the terminology and the phrasing of complex questions, other than clarifying the objectives of the study and the way we would have handled data. Finally, we fixed all typos mentioned by the pilot testers. Upon completion of these modifications, the pilot testers double-checked our work, confirming that their issues were satisfactorily addressed. The detailed suggestions we received are reported in the online appendix of this paper (Ferrara et al. 2022).

### Survey administration and responses

Our empirical study aimed at collecting information from professionals working in the machine learning field, with a focus on fairness-critical projects. In particular, our target practitioners were:

- Software Engineers;
- Data Scientists;
- Data & Feature Engineers;
- Junior or Senior machine learning engineers;
- Junior or Senior Managers of machine learning systems;

We aimed at collecting responses from at least 100 participants, as this number is comparable to similar empirical studies in the field of software engineering (Rafi et al. 2012; Palomares et al. 2017; Garousi et al. 2017), hence increasing our confidence with the conclusions that we may have drawn. One of the key choices for the survey administration was the platform to use. We decided to exclude all social platforms, as they granted us limited control over the participants accessing the survey. We identified Prolific^1^ as a valid alternative for large-scale administration. However, Reid et al. (2022) reported that at least 33% of submissions obtained via a Prolific survey are invalid. To limit the number of invalid answers, the authors recommended to (1) pre-screen participants through the inclusion of background questions aiming at verifying the actual adherence of the respondents to the survey expectations; and (2) add attention checks within the survey, so that the level of engagement of the participants could be measured. We followed those recommendations while designing the survey and, upon collection of the answers, applied a data quality assessment to remove noisy data - more details in Section 4.1. In addition, we limited the survey visibility only to the practitioners that met the following Prolific built-in filters:

- Fluent knowledge of English;
- Work Sector: Information Technology, Science, Technology, Engineering or Mathematics;
- Experience and motivation at work: Excited and highly motivated;
- Study level: Diploma or higher;
- Required skills in computer programming;
- Required knowledge of data science-related programming languages.

Since Prolific did not allow us to set finer-grained filters to select professional figures like Data Scientists, Software Engineers, and similar roles, we explicitly formulated a textual disclaimer that kindly invited users without experience in machine learning engineering and fairness not to engage with the survey. Furthermore, to mitigate the possible self-selection or voluntary response bias, i.e., people who volunteered to respond may be more involved with fairness than the average practitioners, we introduced a monetary incentive of 3.88 USD. Incentives are well-known to mitigate self-selection or voluntary response bias, other than increasing the response rate, as shown in previous studies targeting the methods to increase response rate in survey studies (Avdeyeva and Matland 2013; Church 1993; Smith et al. 2019).

As practitioners might have still ignored the disclaimer and conducted the survey, we planned a follow-up analysis of the answers received, as discussed in Section 4.1. We kept the survey open for a total of 21 days, specifically from May 15 to June 5, 2023.

## Data collection and analysis

### Data quality prescreening

During the period in which the survey was available for respondents to participate, we gathered a total of 197 submissions, which we afterward validated, checking the integrity and consistency of the answers. First, we removed the answers of 20 participants who explicitly declared to have no professional experience with the development of machine learning systems. Of the remaining 177 responses, 49 were considered unreliable and removed from the collected dataset, in particular:

- Six practitioners did not provide enough personal background details to prove their experience in machine learning development;
- 32 practitioners explicitly declared to never worked on fairness-critical machine learning projects;
- 12 participants failed one or both of the attention checks and provided lazy non-sense answers to open-ended questions.

Afterward, we manually inspected the responses, removing 10 more submissions in which we found unreliable responses to the open-ended questions, i.e., answers which were probably generated with a Large Language Model, e.g., ChatGPT. As a consequence of these data-cleaning activities, we finally had 117 submissions filled in by trusted practitioners.

### Analysis strategies

We designed our survey to include both closed-ended questions—in the form of check-boxes, multiple choice combo-boxes, and 5-point Likert scale (Nemoto et al. 2014)—, and open-ended questions.

As the first step of our analysis, we elicited the main patterns through the use of descriptive statistics. For each **RQ** we specifically performed two levels of analysis: (1) We analyzed the general distribution of responses taking into account all valid responses; and (2) We split the responses received according to the counfounding factors discussed in Section 3.2 to assess whether the general trends were still verified when considering subsets of the data.

To extract information from the open-ended questions, we performed content analysis, i.e., a method that allows deriving generalizations from the qualitative data collected (Haggarty 1996). In particular, we followed the main steps of a formal *Deductive Content Analysis* process (Mayring et al. 2004). First, we mapped each open-ended question to a specific Research Question; then, for each question of the survey, we analyzed all answers, summarizing and labelling common information, and finally, we formalized and validated our results.

The first author conducted the data pre-screening and analysis activities and was directly supported and supervised by the second author. The other authors were directly involved in any case of disagreement and to obtain a formal validation of formalized results at the end of the entire data analysis process. It is worth observing that we released the anonymized dataset of responses, the detailed analysis scripts, and results in the online appendix of this paper (Ferrara et al. 2022).

## Analysis of the results

This section overviews the main results of our study. For the sake of readability, we first present information about the participants’ background and then split the discussion by research question.

### Participants’ background

Before discussing the results of the survey study, it is worth reporting on the participants’ background (the complete and detailed data are available in the online appendix of this paper Ferrara et al. (2022)). All the 117 practitioners explicitly declared to have experience in machine learning development and fairness aspects. Most of the respondents (76, i.e., 65.3%) were 18 to 30 years old, while 37 practitioners declared to belong in the age range going from 31 to 50, and 4 people reported to be over 50. As for their level of experience, 51% of the respondents informed us that they have worked for one to three years in such roles, 47% reported four to ten years of experience in their current employment, and only 2 practitioners declared to be in their first year of work. Regarding their current company, 60% of the practitioners declared working in a company with a workforce ranging from 10 to 1,000 employees. Additionally, 38 practitioners declared working in companies with a size of 1,000 to 5,000 employees or larger. The remaining 7% of participants either worked in small environments with fewer than 10 employees or preferred not to indicate this information. A number of 71 out of 117 practitioners qualified themselves as Software Engineers, 38 declared themselves as Data Scientists, 22 as Managers and Project Managers but also other professional figures like Data Engineers , Software Analysts, and Software Architects were well represented. It is worth clarifying that 67 out of 117 practitioners declared to cover more than one professional role. Regarding professional seniority, 69% of the practitioners declared to be employed as junior or senior employees, 18% to hold managerial positions, and another 11% to be self-employed. It is worth pointing out that over 90% of involved practitioners hold bachelor’s or higher degrees, and under 2% preferred not to declare their working seniority.

#### Experience in dealing with ML fairness

Concerning specific experience in dealing with machine learning fairness, 18 practitioners declared limited familiarity with projects that require dealing with machine learning fairness as a crucial aspect. On the contrary, 53 practitioners declared to be skilled or highly experienced in such projects, while the remaining 46 reported having an intermediate experience level. Regarding specific application domains, 65 practitioners declared greater confidence in managing machine learning fairness within technical domains, such as computer vision, networking, or information systems. The remaining 52 practitioners could be considered evenly distributed among various application domains, including economic, legal, and political domains (17 practitioners), medical and scientific domains (14 practitioners), and socio-cultural domains, encompassing education, social media, or literature (21 practitioners).

Regarding specific machine-learning tasks, 67 practitioners stated that they have dealt with fairness in classification problems, while at least 25 practitioners have encountered fairness challenges in typical natural language processing (NLP) tasks, such as speech recognition, machine translation, or data generation and summarization. Additionally, from 30 to 50 practitioners addressed fairness in unsupervised machine learning tasks, such as clustering, anomaly detection, learning ranking, matching, and data generation. It is worth noting that, 73 out of 117 practitioners provided us with additional details about typical examples of fairness treatment in their work experience. In the online appendix, we organized their open-ended feedback to the best of our ability, grouping it based on the primary machine-learning domain or task, as well as specific task or application domains, along with any supplementary information provided (Ferrara et al. 2022).

### RQ_1_ - Definition of fairness in industrial contexts

**Fig. 4:**
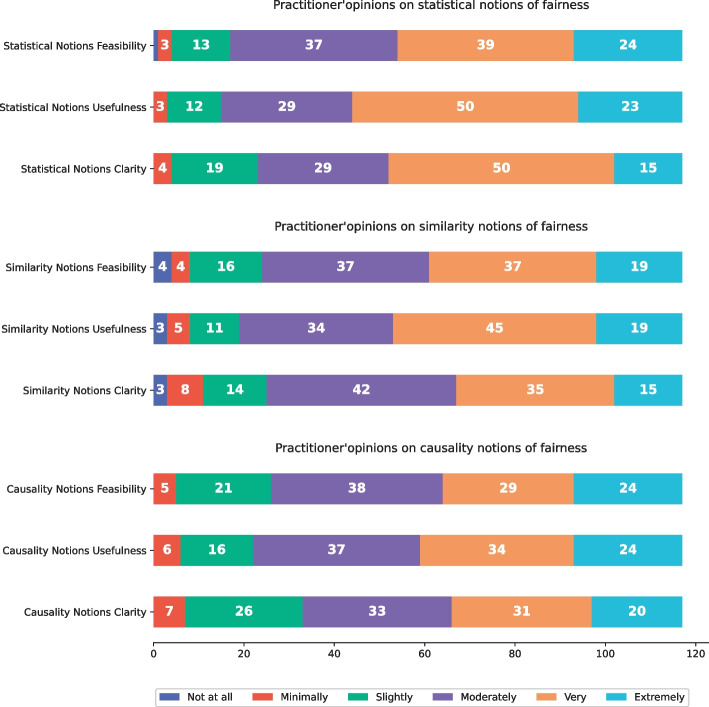
Practitioners’ opinions on the main categories of fairness notions

To deeply understand the difference and similarities among the ways to define machine learning fairness between literature and working practice, we asked to evaluate the three main categories of fairness notions formalized in literature, i.e., statistical, similarity, and causality-based ones, according to indicators we have introduced in the methodological section of this paper (Section 3). Figure 4 reports the main distribution of the general opinions of the practitioners in terms of notion clarity, usefulness, and feasibility for each category of notions of fairness. In addition to the general results, we tried to analyze the distribution of each evaluation indicator in terms of two well-defined co-factors, i.e., the level of education and the size of the company of the respondents, without observing any meaningful difference compared to the general distribution.

First of all, we observed that over 72% of the participants were already familiar with all the main categories of fairness notions, i.e., 86 out of 117 participants adopted at least a statistical, a similarity-based, and/or a causality approach to define and manage fairness in a real working ML-projects. In terms of clarity, i.e., the degree of understandability of a category of fairness definitions, and usefulness, i.e., the improvement gained by using them, the statistical notions of fairness are judged as the better ones (at least 65 high or very high opinions for both indicators), demonstrating that *practitioners preferred to reason in terms of group-fairness definitions to mitigate machine learning disparities*. In addition, similarity-based notions, a.k.a. individual notions of fairness, were considered highly useful by 64 out of 117 participants.

A little different consideration can be observed with respect to the difficulty level in adopting a certain category of notions of fairness in practice. In detail, for all categories of notions, the number of practitioners that consider it highly feasible to put in practice fairness notions are the same that consider it as slightly or moderately feasible, independently from any specific co-factor, e.g., education level or company size. In general, practitioners considered statistical notions of fairness as slightly more applicable with respect to other kinds of notions.

#### Further insights complementing RQ_1_

Beyond the opinions shared via the closed-ended questions, several practitioners also provided answers to the open-ended questions, allowing us to complement the observed results with a deeper understanding of their perspective on the different definitions of fairness.

##### Statistical notions of fairness

Most practitioners believe that statistical notions of fairness are very helpful to address potential biases and discrimination to ensure an equitable outcome in the decision-making process. At the same time, practitioners pointed out that *“the application of fairness statistical notions can vary depending on the specific context and domain”*. For instance, respondents observed that in the contexts of criminal justice, loan approval decisions, hiring, education, social media, and healthcare *“[fairness statistical notions] help analyze the impact of algorithms on different demographic groups”* and *“can be implied to monitor fairness across diverse populations”*. Furthermore, practitioners observed that specific statistical tests can be implied to evaluate and mitigate biases connected to specific sensitive features: *“It’s important to ensure that outcomes are not influenced by things such as race, gender, or religion. For example, statistical tests like the chi-squared test or the t-test can be employed to assess whether there are significant differences in outcomes across different demographic groups”*.

##### Similarity-based notions of fairness

Practitioners stressed the need to involve similarity-based definitions of fairness in *“any use case where the model is unaware of any attributes that are considered to be unfair”*, where identifying correlations among similar individuals is crucial to avoid discriminative predictions. Practitioners are particularly aware of the fact that *“determining what attributes are relevant and how to measure similarity can be complex and context-dependent, requiring careful consideration and analysis”*, and in some cases, the unfeasibility of applying those kinds of notions is connected to *“the availability of the data, but also to the priorities of the stakeholders”*. Nevertheless, when it is possible to properly identify sensitive attributes and similarity functions, these kinds of fairness notions are still widely used to manage fairness in different context-specific issues, e.g., *“to ensure that the distribution of students admitted to a college is the same for all races”* in the context of education, or *“ensuring fair opportunities in job hiring, recommending people not relying on personal preferences, such as hobbies, interests, and backgrounds”*. Practitioners finally provided other domain-specific examples, for example, in the contexts of criminal justice and credit scoring, but also observed how fairness similarity-based notions can be used for more complex tasks like *“image and video analysis where the similarity between images is measured based on visual features like colour, texture, or shape”*, or *“text mining, clustering, text classification, and information retrieval”*, where *“text similarity measures, such as cosine similarity or Jaccard similarity, can be used to assess the similarity between documents or text snippets”*.

##### Causal notions of fairness

Despite the complexity of putting in practice these kinds of fairness notions, due to the complexity to compute a causal graph, most practitioners agreed that they can be implied to *“identify and mitigate sources of unfairness, by examining the causal relationships between variables”*, and provided different domain-specific examples of their usage. For example, in the context of healthcare, causal inference approaches can be used *“to examine the impact of different interventions or treatments on health outcomes across different demographic groups”*, and in the context of credit scoring, *“by analyzing the causal relationships between variables such as credit history, income, and demographic characteristics, researchers can identify the mechanisms through which biases are introduced and develop fairer scoring methodologies”*. In addition, a respondent pointed out that notions like *Counterfactuals Fairness* cannot be used to explain model decisions, since *“a counterfactual identifies an input feature and gives a target input value that the end user should seek to use if they want a different decision”*.

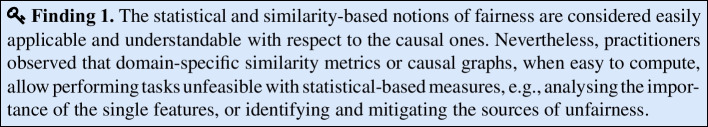



### RQ_2_ - On the relevance of software fairness in the development process

**Fig. 5:**
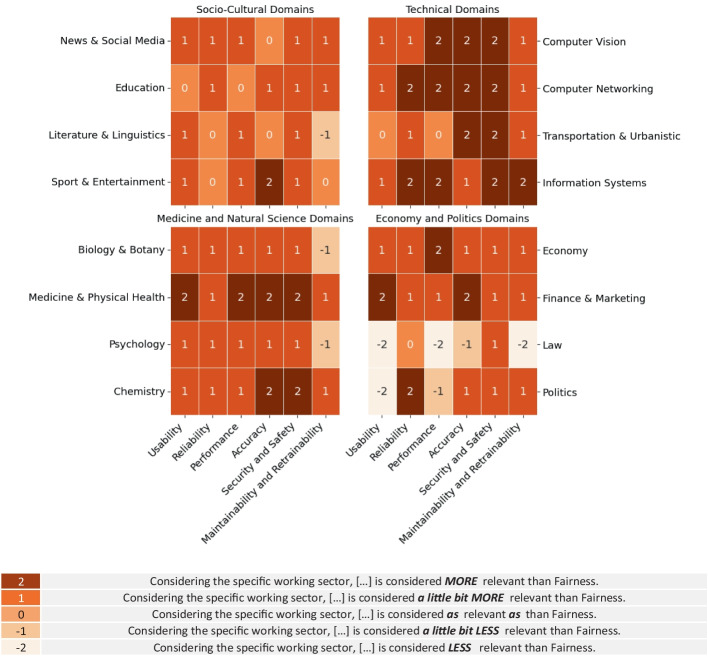
Fairness Trade-Offs Relevance By Sectors

Figure 5 provides an overview of practitioners’ opinions about fairness compared to other quality aspects in a machine learning environment. We structured the main results into four heatmaps, each representing one of the domain areas defined in the methodological part of this study. These heatmaps show the practitioners’ opinions for each comparison between fairness and the other six quality aspects in each of the 16 application domains.

It is worth noting that the specific values refer to the majority of the responses we received for each quality trade-off. For example, if the majority of practitioners considered accuracy more relevant than fairness in the field of psychology, we assigned a value of 2 to the respective cell of the heatmap. Despite the majority of the responses highlighting how fairness is considered a second-class property in the majority of the sectors that practitioners declared feeling more confident in, some exceptions are observable in each of the four domain areas:

#### Socio-cultural application domains

Starting from the socio-cultural domains, it is possible to observe that the 21 practitioners, who expressed opinions in this domain area, considered fairness as **a little less important** than the other quality aspects. However, there are 7 cases in which fairness is considered as relevant as other quality aspects. For instance, the comparisons between fairness and reliability in the fields of sports & entertainment or literature & linguistics.

#### Technical application domains

For this specific domain area, we collected opinions from 65 out of 117 practitioners. It was observed that in almost all cases, practitioners considered fairness as slightly **less important or strongly less important** than other non-functional properties. However, it is interesting to observe how the majority of the respondents retained the usability and the performance of the system as relevant as fairness in the field of transportation & urbanistic.

#### Medicine and natural science domains

14 out of 117 practitioners provided us with opinions in this specific domain area. To the best of the responses we received, it is possible to observe that **practitioners considered fairness as a second-class non-functional aspect for almost all of the non-functional trade-offs**. It is worth observing that the practitioners considered fairness as **strongly less important** than the majority of the other non-functional properties in the field of medicine & health, but **a little bit more important than** maintainability and retrainability in the fields of psychology and biology & botany.

#### Economy and politics

Similarly to the previous domain area, we collected 17 opinions out of 117 for the quality comparison for the economic, legislative, and political application domains. We obtained similar insights achieved for the scientific domains in the contexts of the economy and finance. However, fairness is considered **more important than usability and system performance** in the field of politics and law, and **more important than** model accuracy, maintainability, and retrainability in the law application domain.

It is worth noticing, that in this paper we reported only the main trends of the practitioners’ opinions about the single trade-offs, while the detailed responses are available in the online appendix (Ferrara et al. 2022).

#### Further insights complementing RQ_2_

Discussing their opinion about fairness’s relevance with respect to other non-functional properties of ML systems, practitioners observed how the trade-off between fairness and model accuracy is widely monitored during the development process. They also shared specific strategies to balance those aspects with respect to the domain specificity of the project. For instance, in the context of hiring, a practitioner pointed out that *“one approach to balance fairness-accuracy trade-off is to incorporate fairness constraints in the model’s optimization process. For instance, organizations can aim to achieve a certain level of fairness while maintaining a reasonable level of accuracy”*. Another interesting strategy that practitioners suggested is to imply multi-objective optimization approaches, observing how improving the fairness of a decision-making system, can positively reflect on its level of accuracy, e.g, a practitioner in the context of loan approval observed that *“a more accurate model may rely on historical data that reflects biased lending practices, resulting in potential discrimination. Balancing fairness, such as ensuring equal approval rates across different demographic groups, may require sacrificing some level of accuracy”*.

Furthermore, practitioners suggested that fairness can influence the model explainability results, especially when models are complex, i.e., deep neural networks. They observed that *“in such cases, simpler and more interpretable models or techniques like rule-based models or decision trees can be preferred to ensure fairness and maintain transparency”*. They also provided useful insights about other non-functional aspects that could be affected by fairness, such as user privacy in the context of healthcare, *“where balancing fairness and privacy is crucial, and sharing sensitive medical data for training models can improve fairness, but it raises privacy concerns”*, or model efficiency in the context of bioinformatics, *“where systematic bias is well-known, and often occurs in the high-throughput processing of microbiological specimens for testing, due to small systematic differences in the machines or environmental condition”*.

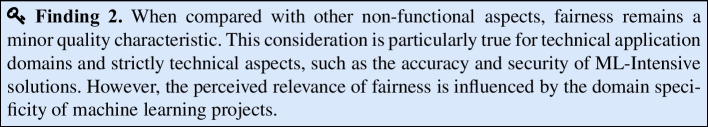



**Fig. 6:**
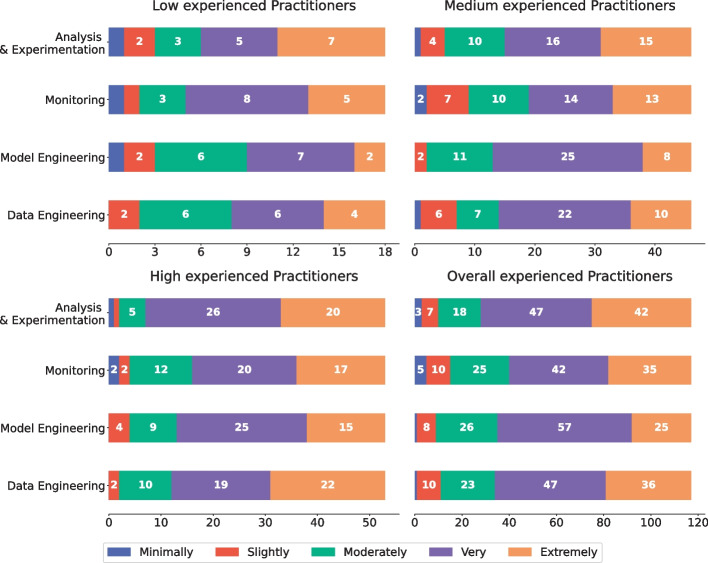
Relevance of fairness at different stages of a typical ML pipeline

**Fig. 7:**
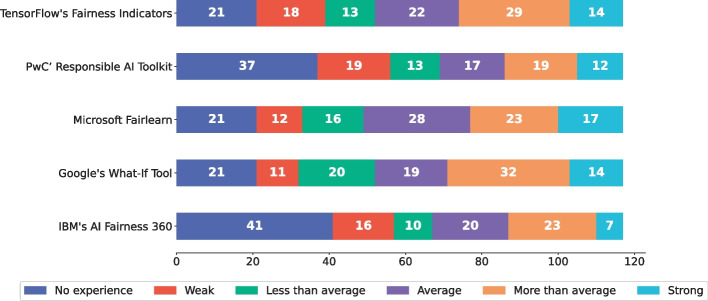
Practioners’ degree of experience with specific fairness management tools in an ML project

### RQ_3_ - On the engineering of software fairness within an MLOps pipeline

RQ_3_ focused on evaluating whether and in which phases of an ML pipeline it is relevant to adopt strategies to deal with software fairness.

Figure 6 presents the participants’ opinions regarding the usefulness of applying fairness treatment practices in each step of an ML pipeline. For all the steps, more than 75% of the practitioners declared that employing strategies to deal with fairness is worthwhile. In detail, 89 practitioners considered the *Data Analysis and Dataset Experimentation* phase as the most relevant when fairness is a crucial aspect, while the *Model Engineering* phase was slightly less relevant than the other three phases. Additionally, we also included the opinions of the practitioners grouped by their level of experience in dealing with machine learning fairness, but no significant differences were found compared to the general distribution.

Based on the results, we identified the need for novel methodologies and processes, in addition to metrics and tools, to handle fairness throughout the lifecycle. This highlights the relevance of data management and improving adherence to specific fairness constraints.

In addition to providing insights about the phases in an MLOps pipeline where managing machine learning fairness would be useful, the practitioners also expressed their opinions on five widely known fairness treatment tools in the literature. Figure 7 shows the practitioners’ level of experience with these tools. We found that at least 60% of the respondents had previous experience with each of the five tools. Specifically, 37 and 47 practitioners declared no previous experience with the *PwC Responsible AI Toolkit* and *IBM’s AI Fairness 360*, respectively, while only 21 practitioners reported no past experience with the other three tools.

As shown in Table 5, we also asked the practitioners to indicate in which phases of a machine learning pipeline such tools might be considered to practically manage machine learning fairness at different stages. On one hand, it is noteworthy that 49 and 47 practitioners found it useful to adopt specific fairness analytics tools, such as *Google What-IF* and the *Tensor Flow Fairness Indicators*, during the operationalizing phase of *data analysis and dataset experimentation*. On the other hand, *Microsoft Fairnlearn*, as an integrated fairness treatment platform, was considered useful by more than 30 practitioners in each stage of the MLOps pipeline.

**Table 5:** Fairness tools usefulness at different stages of a ML Pipeline

	No Idea	Data Engineering	Model Engineering	Performance and Quality Monitoring	Analysis and Experimentation
IBM’s AI Fairness 360	46	23	29	42	24
Google’s What-If Tool	26	26	36	49	34
Microsoft Fairlearn	31	37	32	43	38
PwC Responsible AI Toolkit	46	16	32	36	24
TensorFlow’s Fairness Indicators	29	29	44	47	32

#### Further insights complementing RQ_3_

By answering the open-ended questions, practitioners provided us with insights into the relevance of adopting different pre-processing strategies ensuring representativeness and diversity in data. They suggested that *“it is important to carefully select features and consider the potential impact on fairness”* in order to avoid using sensitive attributes directly as training input and explore alternatives, i.e., fairness-aware feature transformations, or *“implement data augmentation techniques to increase the diversity of the dataset and ensure fair representation”*. Respondents highlighted the importance of introducing specific constraints and adopting specific metrics to properly manage and monitor fairness at training time, e.g., *“optimizing for equalized odds or demographic parity, and regularly evaluate the model’s performance across different demographic groups and assess fairness metrics”*. In addition, some practitioners suggested incorporating user feedback at training time and re-evaluation process in order to *“identify potential fairness issues or biases in the ML system, and conduct fairness impact assessments on updated versions of the model”*.

Practitioners provided additional information about the usage of specific tools to address machine learning fairness in various application domains. With respect to the tools we listed in the questionnaire, the practitioners observed how tools like *Microsoft Fairlearn* and *IBM’s AI Fairness 360* *“offer functionalities to assess fairness and implement techniques like reweighing, fairness-aware training,”* that are widely used to remove bias in the development of ML-Intensive solutions for resume prescreening, credit scoring, and ats widely used to *“analyze the predictions made by the advertising model across different demographic groups, helping to explore model’s behaviour varies for various subsets of the population, such as age, gender, or race”*.

In addition, practitioners suggested other fairness-specific tools used in different application domains, e.g., (1) the *Aequitas* tool (Udeshi et al. 2018), *“that provides various fairness metrics and algorithms to measure and mitigate bias in criminal justice applications”*, as well as fairness-aware post-processing algorithms *‘such as the Prejudice Remover Regularizer or Equalized Odds Post-processing”*, useful to ensure fairness in risk predictions and other decision-making processes, and (2) *Themis-ML* (Bantilan 2018), *“an open-source Python library that offers fairness metrics and algorithms for mitigating bias in healthcare applications”*.

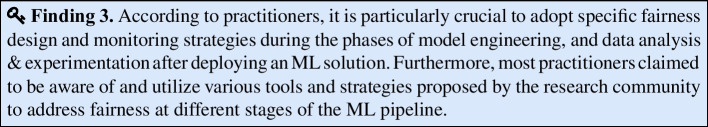



### RQ_4_ - On the team composition for the development of fair ML-intensive systems

#### Machine learning pipeline

**Fig. 8:**
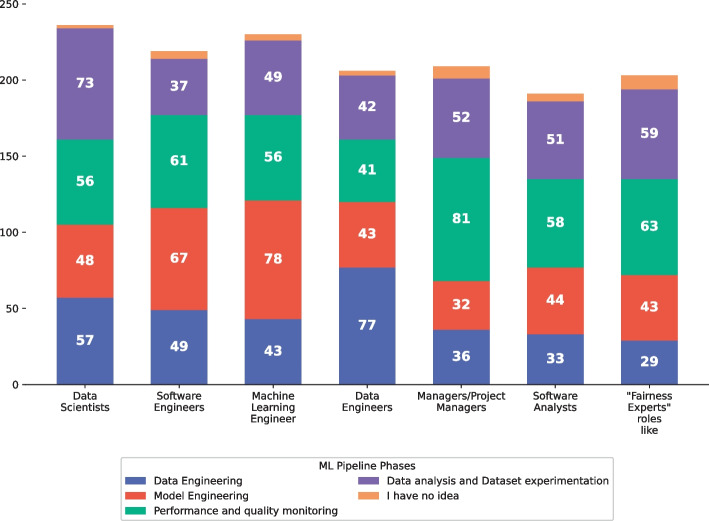
Professional roles required to manage fairness at different stages of an ML pipeline

The diagram in Figure 8 depicts practitioners’ opinions on the professional roles accountable for the fairness of a machine learning system. Specifically, the diagram indicates the specific roles that are particularly necessary at each stage of development to handle ethical aspects throughout the entire life cycle of an intensive ML system. Starting with the data engineering phase, it is observed that 77 practitioners believe that the role of a data engineer is essential in feature selection and bias detection. They are directly supported by technical roles such as data scientists (57 practitioners), software engineers (49 responses), and machine learning engineers (43 responses). This result confirms the professional significance of initial data preparation for proper fairness management and highlights the need for active collaboration between data science and software engineering roles. Regarding the Model Engineering phase, practitioners consider the presence of engineering roles crucial in model and algorithm selection to prevent discrimination in the operational stages of a machine learning pipeline. This is evident from the 67 practitioners who indicate the role of a software engineer in this phase of the life cycle and the 78 responses related to the specific role of a machine learning engineer. Other notable considerations revolve around the relevance of project managers in monitoring and ensuring the ethical quality of the developed model. 81 practitioners indicate the significance of managers in addressing fairness in this specific development phase. They are directly supported by several involved roles, such as software engineers (61 respondents) and experts in ethics and fairness (63 responses). In the end, ethics and fairness experts have been considered highly relevant in the operational phase of data analysis and experimentation (59 responses), working closely with data scientists (73 practitioners). As for the previous research questions, we provided fine-grained results by dividing the overall data by the company size of the practitioners; the additional analysis, available in the online appendix (Ferrara et al. 2022) confirms the trends we have discussed, especially in the context of medium and large companies.

**Fig. 9:**
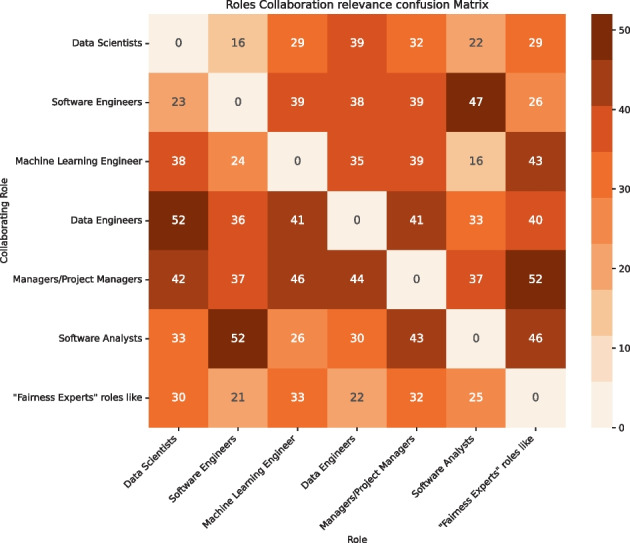
Collaborations between professional roles required to manage fairness in a typical ML project

In addition to asking which professional roles have the most impact on managing fairness in a machine learning pipeline, we also obtained insights from industry experts on “which collaborations and interactions between different professional roles are particularly impactful in effectively managing fairness in an intensive ML project”. As shown in Figure 9, it is particularly crucial to have extensive interactions between managers and ethics/fairness experts (52 out of 117 practitioners). Additionally, collaborations between managers and machine learning engineers (46 responses), data engineers (44 responses), and data scientists (42 responses) are also deemed significant for fairness treatment. These findings regarding the importance of specific roles in the pipeline suggest, the significance of timely intervention in detecting and addressing ethical issues in the operational stages of a machine-learning pipeline. Support is needed not only from a managerial perspective but also in terms of the design and selection of appropriate strategies for mitigating such issues. This is further supported by the 52 practitioners who highlight the necessity of collaborations between software engineers and software analysts, who play vital roles in ethical treatment both during the development and maintenance phases of a machine learning project.

#### Further insights complementing RQ_4_

In the open-ended answers, practitioners mentioned how it is crucial to engage and collaborate with multiple stakeholders to address fairness throughout the different stages of a typical machine-learning pipeline. First of all, practitioners suggested engaging in a discussion with ethicists and legal experts *“to identify and evaluate potential ethical challenges and guide the decision-making process and ethical guidelines and ensure alignment with societal values”*. In addition, they pointed out the need to directly involve the domain-specific experts that *“possess deep knowledge of the specific application domain”* necessary to identify potential biases, understand contextual factors, and provide insights on fairness considerations specific to that domain. Furthermore, respondents observed that the expertise of data scientists and machine learning engineers is vital in implementing fairness-aware models. Nevertheless, project and product managers play crucial roles in *“prioritizing fairness considerations, and ensuring that the development process aligns with ethical standards and fairness goals into the product roadmap and decision-making processes”*. External auditors and third parts evaluators *can provide an objective assessment of the fairness of the machine learning system, conducting independent audits, or reviews to validate the fairness practices and identify any biases*.

Practitioners also provided their opinions on collaborative efforts among different professional roles to address fairness in various domain-specific contexts. They provided domain-specific examples about the importance of interacting with domain-specific, legal, and ethical experts. For example, in developing facial recognition technologies for criminal justice, practitioners observed that *“collaborations between ethicists, researchers, data scientists, and legal experts can lead to significant advancements in fairness, identifying and addressing biases related to gender and race in facial recognition algorithms”*. Similar observations were also made with respect to fairness-critical systems for healthcare and autonomous driving. In addition, practitioners also remarked that *“it is important to let the project manager work closely with the fairness expert. With this close working relationship, it is possible to get an early notice for big changes that had to be made”*,

Finally, participants observed that the majority of fairness issues can be detected and managed only by considering a collaborative workflow during the entire machine learning pipeline, with particular attention to the phases of data and model engineering, e.g., in the context of online advertising *“Data Engineers preprocess and prepare advertising data, ensuring that it is representative and unbiased. Data Analysts and Data Scientists analyze the data to identify potential biases in ad targeting and develop a fair ad targeting model”*. Also monitoring models’ behaviour in the late operationalizing steps of a machine learning pipeline is crucial, as *“Performance and Quality Monitoring teams continuously monitor the ad targeting system, assess fairness metrics, and address any biases that may emerge to promote fair distribution and equal opportunities for diverse audiences”*.

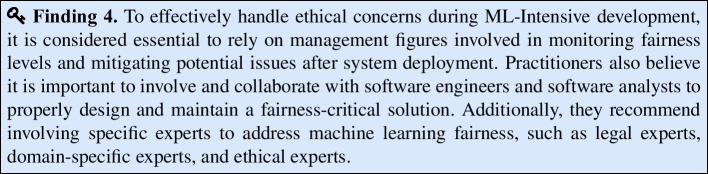



## Discussion & implications

The results of our investigation ignited a number of reflections and discussion points, including implications for researchers and practitioners that can be further explored in future work.

### On the tight connection between fairness and its application domain

Confirming the findings of previous work (Brun and Meliou 2018; Verma and Rubin 2018), our empirical investigation highlighted that the ethical aspects of a machine learning solution are difficult to generalize and treat in a standardized way. Answering RQ_1_, we observed that different fairness definitions and approaches are considered more or less suitable to deal with fairness. Furthermore, we observed that the relevance of fairness changes according to the specific application domain the practitioners work in. We believe that one of the main goals of future research should be the analysis of the correlation between the ethical aspects of machine learning and the specificity of application domains. We conjecture that taxonomies and mapping studies on fairness definitions, metrics, and constraints concerned with domain-specific scenarios can guide practitioners to choose proper strategies to deal with fairness in real-case contexts.

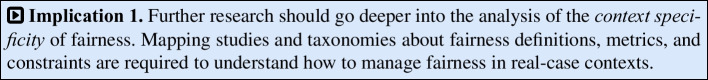



### Toward the standardization of fairness in a machine learning pipeline

The findings of our investigations highlighted that the ethical issues affecting machine learning solutions can be assessed and mitigated in each phase of the pipeline. However, fairness is not perceived yet as a key quality aspect, like accuracy or security. We believe that it is crucial to raise awareness about fairness problems emerging in ML-Intensive systems, by educating practitioners via the application of standardized processes and methodologies. In particular, further effort is needed from the research community to provide detailed guidelines and ready-to-use tools to deal with fairness, from the requirements analysis phase to the model deployment and monitoring. For example, a systematic catalog of best and bad practices to deal with fairness could suggest that fairness should be managed in each step of a machine learning pipeline.

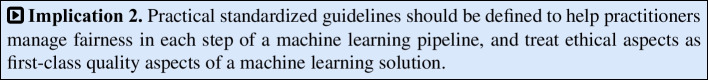



### Defining and managing ML fairness: theory versus practice

Over the years, a number of relevant works have proposed several notions and metrics of fairness, up to create well-structured taxonomies, among the most famous being those proposed by Verma and Rubin (2018) and Mehrabi et al. (2021). As already stated in the literature, probabilistic approaches and definitions are among the most widely used and less computationally expensive. Although such probabilistic definitions turn out to be not easy to understand in the eyes of non-experts (Saha et al. 2020), industrial practitioners find them very useful in quantifying intrinsic aspects of Group Fairness for a wide variety of application domains. Furthermore, practitioners observed that those definitions are widely implemented in different tools, that they adopt in practice in the monitoring and evolution phases of a canonical ML module. At the same time, practitioners pointed out that while there are well-known difficulties in their application, causality, and similarity-based approaches help to analyze the causes of unfairness practically where probabilistic definitions are limiting and not exhaustive. Despite the difficulties in identifying ad-hoc similarity functions for the specific application domain (Mehrabi et al. 2021), and the practical complexity in computing and modifying individual nodes and arcs of causal dependency graphs (Kusner et al. 2017), respondents implicitly suggested that researchers should invest in formalising and design new approaches and tools that practically involve those kinds of solutions.

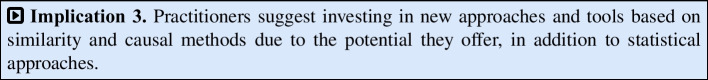

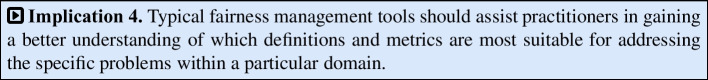





### To what extent can fairness be compared to other quality aspects?

A common practice in the literature is to compare different, often incompatible definitions and metrics of fairness, which, depending on the case, improve or worsen fairness levels, also impacting the performance and accuracy of the system (Pessach and Shmueli 2022). Consequently, *trade-offs* between system fairness and accuracy, as well as fairness and performance, have been defined in the literature, driving modern ensemble learning strategies designed to simultaneously improve multiple qualitative properties of an ML module (Chen et al. 2022; Hort et al. 2021). Among the contributions of our work, we provided a broader view of the fairness-specific trade-offs. Through our survey, we observed that practitioners continue to perceive fairness as less relevant than many other aspects of quality, such as the model’s accuracy, security, reliability, and maintainability. Based on the specific domain of application, it is possible to infer that fairness may be considered more relevant than other aspects, or that practitioners themselves compare other non-functional specific aspects with the ethical aspect, e.g., fairness compared to data privacy in the medical field.

Based on these findings, it is necessary to conduct new studies aimed at expanding and systematizing the perception of fairness with respect to other specific aspects of ML development, especially in relation to the specific application domains.

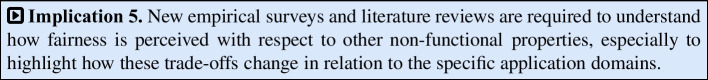



### Towards process-driven approaches for ML Fairness

As an innovative and widely treated topic, different and various studies are conducted in the context of machine learning fairness. Nevertheless, the research community treated this theme only from a product-specific point of view, proposing various resources, such as datasets (Fabris et al. 2022; Le Quy et al. 2022; Catania et al. 2022), metrics and tools (Pagano et al. 2023; Mehrabi et al. 2021), but also possible unfairness causes, i.e., all different kind of biases that can cause discriminations (Mehrabi et al. 2021). One of the main findings of our work is given by the possibility to face and manage fairness issues from a process-driven point of view. Practitioners highlighted that cross-functional professional figures, e.g., managers and engineers, are widely involved in monitoring fairness, especially in the late stages of a machine learning pipeline. We also observed that collaborations among different roles in a fairness-critical development process make the difference to identify, manage, audit, and remove bias. Practitioners observed that domain, ethical, and legal experts are particularly relevant for these kinds of activities. We believe that further investigations with practitioners, such as focused semi-structured interviews, are necessary in order to understand and better organize the treatment of fairness from an engineering perspective, creating standards and meta-models of development that assist practitioners in various context-specific needs.

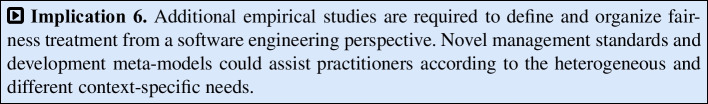



## Threats to validity

This section reveals the possible threats that could affect our empirical study and the strategies we adopted to mitigate them. To discuss such threats, we followed the guidelines proposed by Wohlin and Runeson (2012).

### Conclusion validity

The first set of potential threats to an empirical study concerns the methodologies used for the data analysis and the generalization of results. In particular, our work is affected by the following threats:

- **Reliability of measures**. The design quality of our survey was particularly critical to answer our research questions properly. We defined structured multiple-choice questions, for which the possible answers could be values in a range or multiple-selection values. Considering the qualitative nature of the study, we also included open questions—one for each relevant closed-ended question—through which participants might have provided further insights on the matter.
- **Reliability of treatment**. Still considering that a low-quality survey design could produce unreliable results, we directly included specific filtering instruments, i.e., attention-check questions, that allowed us to conduct a deep quality pre-screening process before the formalization of the results, and alternative flows, that allowed practitioners to respond to domain-specific questions only in relation to their expertise in the field.
- **Sample representativeness**. Another relevant aspect that could have affected the reliability of the results is the level of representativeness that the sample has with respect to the study population or specific subgroups of individuals. In our case, this threat was mainly connected to the domain-specific questions, e.g., comparisons between fairness and other quality aspects in different machine learning application domains. To face this threat, we first collected information on the past experience of the respondents with machine learning fairness and the application domains they were involved in; afterwards, we made sure to ask practitioners to answer only those domain-specific questions related to their own expertise. This increased our confidence in the reliability of answers, and the representativeness of the considered sample to gather insights related to specific application domains.
- **Sample size**. The number of respondents might have affected the reliability of the results. We aimed at collecting responses from at least 100 experienced participants. The overall amount of responses obtained, i.e., 117, is comparable to similar studies in the field of empirical software engineering (Rafi et al. 2012; Palomares et al. 2017; Garousi et al. 2017). In addition, the filters set and the performed quality assessment checks allowed us to rely on practitioners having experience in the development of fairness-critical systems. As such, we are confident that the findings of the paper well represent the more general perspective of practitioners with respect to the engineering of machine learning fairness. Yet, we still acknowledge that in the cases of the domain-specific questions we had to analyze smaller sub-samples: as such, further replications of our work would be desirable to corroborate our findings.

### Construct validity

The construct validity of an empirical study concerns the aspects that can mine the relation between the study hypotheses and observations.

- **Hypothesis guessing**. Short and badly designed survey may cause hypothesis guessing: in response to this threat, we structured our survey contents following an ad-hoc logical flow, in order to insert in the same survey sections questions that are logically related to more than one research question. In addition, we made sure to phrase the survey questions in a way that they would not bias the practitioners’ ideas and responses—in this sense, the survey validation step conducted through the pilot test increased our confidence in the survey design quality.
- **Level of Constructs**. Since we used an administration platform that involved practitioners with different backgrounds and expertise, the results could be affected by the poor quality and comprehensibility of the survey and the skills required to deal with the specific subject under study. To guarantee the highest level of comprehensibility, we formulated all questions in English, avoiding long sentences and technical vocabulary. In addition, we encouraged participation, emphasizing the need for specific knowledge in the areas of Software Engineering or Artificial intelligence and fairness-critical machine learning development.
- **Mono-Method Bias**. Considering that we collected information through a single focused survey to validate contents and duration, we conducted a pilot test with Ph.D. students in Computer Science, which highlighted some criticisms about the survey contents and required time that could have impacted our research findings. We solved the raised issues by clarifying the terminology used, particularly when referring to technical concepts and definitions, improving the phrasing of some questions, and better clarifying the study objectives and the data management policies, other than by fixing minor issues, like typos and/or grammar mistakes.
- **Experience Bias**. Another possible threat to construct validity is connected to the potential misalignments in experience between Ph.D. students who took part in the pilot test (only leveraging theoretical knowledge) and practitioners we surveyed to answer our research questions (benefitting from theoretical knowledge augmented by experience). As such, practitioners may have taken less time to complete the study due to their experience, or perhaps more time given the higher level of detail they were able to provide. To partially mitigate this threat and ensure that the required time of completion measured in the pilot test was in line with the actual completion time taken by the practitioners in the survey, we involved five Ph.D. students working on research themes connected to machine learning engineering and software fairness—one of them also had a three-year previous working experience as a data scientist. The reliance on those Ph.D. students allowed us to mitigate the risk of misalignment between pilot testers and our target population.
- **Time-Efficiency Bias**. Through the pilot test, we estimated that the mean time required to complete the survey ranged between 15 and 20 minutes; extreme outliers of such a range might have indicated participants not taking the task seriously, hence potentially biasing our results. To avoid errors in the estimation, we assessed post-execution of the survey that the actual mean time of completion by practitioners was close to 20 minutes. In addition, we manually verified the reliability of all submissions received, paying special attention to the responses coming from practitioners who took too much or too little time.

### Internal validity

Internal validity could be mined by a homogeneous sampling or by participants with poor experience in the ML-Intensive development field. The platform chosen for our study allowed us to directly interact with the target population, mitigating a number of potential threats:

- **Selection**. In an empirical investigation, people recruited could negatively influence the study if demotivated or not adequately rewarded, other than introducing self-selection or voluntary response bias. Under Prolific policies, the participants were recruited on a voluntary basis and encouraged through a financial reward; this reward can be seen as an incentive. Incentives are well-known to increase the response rate, as shown in previous studies targeting the methods to increase response rate in survey studies (Avdeyeva and Matland 2013; Church 1993; Smith et al. 2019)—the employed online recruitment platform itself, i.e., Prolific, is built upon such observations.
- **Incentive Bias**. The use of rewards may even become detrimental, as they may introduce the possibility of systematic bias in how practitioners respond. As recommended by previous work (Ebert et al. 2022), we mitigated this risk through the implementation of multiple strategies, including (1) the specification of exclusion criteria through the Prolific built-in filters, which allowed us to filter out individuals who did not meet the minimum requirements for participation, i.e., motivation at work, fluency in English, ability to work with data science-related programming languages, and employment in the computer science field(s); (2) the presence of attention checks, which may test the participants’ attention, hence possibly spotting cases where participants answered the survey questions just to obtain a reward, without providing detailed feedback; and (3) a data quality assessment made upon survey completion, where we manually verified the time spent by each participant on the survey and the quality of the responses provided, in an effort of spotting cases where participants neglected the task. While we cannot estimate the number of participants filtered out because of strategy (1)—the Prolific built-in filters only provide an estimation of the prospective users who may participate in the survey—over 45% of the responses were manually removed upon completion as a consequence of the application of strategies (2) and (3). In other terms, we did our best to exclude answers characterized by evident poor relevance and quality and build our work upon reliable opinions. We are aware that some bias due to incentivizing participants can be still present, which is why we call for replications of our survey study.
- **Mutation & History**. Considering that the administration phase of our study required more than one day to be completed, the consistency of the collected information could be affected during the administration period. We recognized the necessity to constantly monitor the evolution of the gathered data, therefore we periodically checked the received responses according to our quality pre-screening choices.

### External validity

External validity regards the generalizability of the research results.

- **Limited Generalizability.** Our results and the conclusions drawn in our study are strictly related to the sample, hence being potentially affected by limited generalizability. When conducting large-scale survey studies concerning domain and task-specific topics, like machine learning fairness, it is critical to gather opinions from experienced practitioners having different backgrounds and working in different domains. As a consequence of the selection procedures employed and the quality checks performed, we may argue that our findings reasonably reflect the opinions of practitioners involved in multiple fairness-critical application domains and engaging with different machine-learning tasks. However, we cannot claim that our results hold for the largest population of machine learning engineers working on fairness-critical domains that were not represented in the sample. Additional studies would be needed, particularly investigating possible differences and implications in underrepresented application domains.
- **Professional Environment Effect.** Another factor possibly impacting the generalizability of the sample may be connected to the professional environment participants are involved in. For instance, companies implementing explicit strategies or policies to deal with fairness might train their employees on the matter; this might result in practitioners being more aware of the problem, its impact, and the practices to apply to deal with it. We did our best to collect background information that may properly describe the sample taken into account in our study; nonetheless, we still acknowledge that some external factors may have affected our conclusions. Our study may be therefore considered as a baseline for further investigations into the matter. On the one hand, replications in specific application domains not targeted by our work might be beneficial to extend the knowledge of how fairness is managed in practice. On the other hand, researchers working in the field may complement and broaden the current body of knowledge through different research methods, e.g., software repository mining analyses of how machine learning engineers deal with fairness requirements.
- **Geographical Dispersion.** Looking at the demographics of our survey participants, we could observe that most of them were from Europe. As such, our results may be biased toward the working habits of Europeans. To assess this potential threat to validity in the context of our study, we performed an additional analysis only considering the answers provided by non-European practitioners to see whether they were consistent with the general patterns observed. We found a significant consistency which allows us to argue that Europeans’ opinions indeed reflected a larger population’s view. However, we are aware that replications of the study targeting a more variegate socio-cultural and geographical background would be beneficial to corroborate our findings.

## Conclusion

This paper focused on the practitioners’ perspective on software fairness and how it is treated in practice. The shared vision of the research community in the Software Engineering for Artificial Intelligence field suggests that fairness is difficult to generalize and needs new research studies that cover all main aspects of an ML pipeline. To verify whether practitioners effectively treat fairness during the development, we conducted a large-scale survey to gather information about (1) the degree of clarity, usefulness, and feasibility of the main category of notions of fairness, (2) the relevance of fairness compared to other quality aspects of a system, (3) the applicability of specific fairness treatment strategies and tools in an ML-Intensive pipeline, and (4) the composition of a team working on ML-Intensive fairness-critical solutions. Our results show how fairness needs new research studies before being considered a first-class aspect as mature as other quality attributes of an ML-Intensive solution. Practitioners prefer to formally define fairness using statistical-based notions, but also causal reasoning and similarity-based are taken into account. We observed that fairness should be treated in particular evolutionary steps of an ML pipeline, i.e., during the phases of Data Preparation and data analysis & experimentation after deployment. Practitioners highlighted that cooperation among cross-functional roles like Data Scientists and Software Engineers and the direct involvement of Managers and Fairness Experts, i.e., ethical, legal, and domain-specific experts are crucial for achieving proper levels of fairness. It is worth remarking on how practitioners’ perspective is crucial to make fairness a *first-class* quality aspect in the machine learning field. We believe that new Software Engineering studies, such as empirical analyses on fairness treatment practices, are crucial to enhance the understanding of fairness, eventually leading to the definition of novel ad-hoc approaches to deal with fairness.

When it turns to our future research agenda, we aim at corroborating our conclusions by means of further replications conducted using samples with different characteristics with respect to those considered in our study, other than through different research methods, e.g., interview- or focus group-based research methods, which would be beneficial to further increase the knowledge on the current state of the practice related to machine learning fairness.

## Data Availability

This paper includes data as electronic supplementary material. Datasets generated and analyzed in the context of this study, raw results, and detailed plots, as well as additional resources useful for reproducing our research, are available in the online appendix of this paper: http://doi.org/10.6084/m9.figshare.21680519.

## Footnotes

1 The Prolific platform: https://www.prolific.co.
